# Masticator space abscess mimicking a malignant tumor

**DOI:** 10.11604/pamj.2024.47.103.42765

**Published:** 2024-03-04

**Authors:** Sameh Mezri, Oumaima Zitoun

**Affiliations:** 1 Ear, Nose, and Throat Department, Military Hospital of Tunis, Tunis, Tunisia

**Keywords:** Masticator space, infection, radiology

## Image in medicine

Differentiation of masticator space affections may be difficult regarding the complexity of the anatomy and the access. Infection may be a serious condition due to the risk of extension in the upper aerodigestive space and the infratemporal fossa. Magnetic resonance imaging (MRI) with ADC study can help to discriminate differential diagnosis. A 59-year-old male presented a right cheek swelling progressively increasing in size for a month under ten days of amoxicillin-clavulanic acid therapy. Physical examination revealed a firm, ill-defined, and painful mass in the parotid region, with severe trismus. The parotid duct and salivary flow have normal physical aspects. The patient had facial paresis with hypoesthesia but no cervical lymphadenopathy. A biologic test showed a C-reactive protein level of 24.5 mg/dL. Contrast-enhanced CT (A) showed a cystic formation within the masticatory space centered on the lateral pterygoid muscle and the temporal muscle, with lateral extension encompassing the masseter muscle. Malignancy was suspected. MRI showed an expansive lesion process developing in the right masticatory space, with extended heterogeneous signal in the masseter, temporal, and lateral pterygoid muscles, including internally enhanced necrotic zones, also extending to the pterygo-palatine process. (B, C, D). We underwent a surgical exploration and we have a discharge of pus. A bacteriological sample was negative. A biopsy of the surrounding tissues concluded with an inflammatory remodeling of the masseter muscle. The evolution was marked by the normalization of clinical and radiologic signs under 14 days of Cefotaxime and Metronidazole.

**Figure 1:**
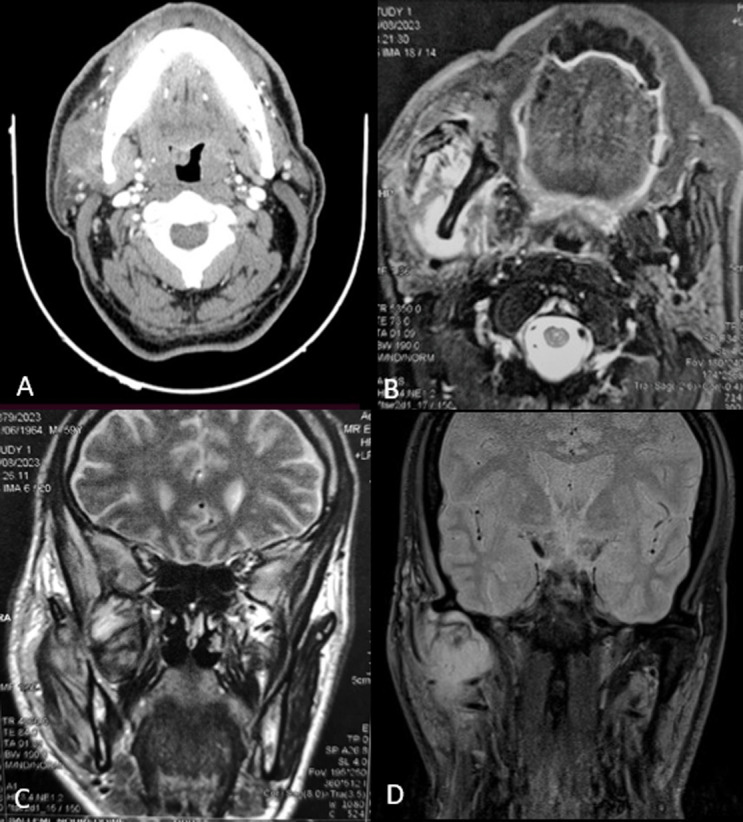
A) contrast-enhanced CT showing a cystic formation within the masticatory space centered on the lateral pterygoid muscle and the temporal muscle, with lateral extension encompassing the masseter muscle; B, C, D) MRI showing an expansive lesion process developing in the right masticatory space, with extended heterogeneous signal in the masseter, temporal, and lateral pterygoid muscles, including internally enhanced necrotic zones, also extending to the pterygo-palatine process

